# Bibliometric and visual analysis in the field of the ketogenic diet in relation to brain health from 2013 to 2024

**DOI:** 10.3389/fnut.2025.1479699

**Published:** 2025-03-19

**Authors:** Yuanyuan Yan, Yuanchu Lian, Zonghuai Li, Bo Zhang, Pingping Liu, Guihong Huang

**Affiliations:** ^1^Department of Pharmacy, Sanya Central Hospital (The Third People’s Hospital of Hainan Province), Sanya, China; ^2^Scientific Research Center, Guilin Medical University, Guilin, China; ^3^Department of Pharmacy, The Second Affiliated Hospital of Guilin Medical University, Guilin, China; ^4^Guangxi Health Commission Key Laboratory of Glucose and Lipid Metabolism Disorders, Key Laboratory of Diabetic Systems Medicine, The Second Affiliated Hospital of Guilin Medical University, Guilin, Guangxi, China; ^5^Guangxi Key Laboratory of Drug Discovery and Optimization, Guangxi Engineering Research Center for Pharmaceutical Molecular Screening and Druggability Evaluation, School of Pharmacy, Guilin Medical University, Guilin, China

**Keywords:** bibliometric, ketogenic diet, brain health, metabolism, oxidative stress, neuroprotection

## Abstract

**Objectives:**

The metabolites of the ketogenic diet (KD), specifically ketone bodies (KB), are closely linked to brain health. The KD is widely used to treat epilepsy. It’s also getting more attention for treating neurodegenerative disorders like Alzheimer’s and Parkinson’s diseases, and its effectiveness in these areas is well - recognized. This study aims to explore the research hotspots in the field of KD and brain health from 2013 to 2024, providing references and directions for future research.

**Methods:**

This study utilized R software, VOSviewer, and CiteSpace to analyze 1,162 publications in this field from 2013 to 2024.

**Results:**

A total of 1,162 publications were included in this study. From 2013 to 2021, there was an upward trend in the number of publications in this field, followed by a slight decline from 2021 to 2023. The United States has the highest number of publications and exhibits the most extensive collaboration with other countries, positioning it as the leading nation in this field. The journal Nutrients has the highest number of publications, while Epilepsia is the most cited journal. Key subject terms include KD, Brain, Beta-Hydroxybutyrate, KB, Metabolism, and Oxidative Stress. The primary research focuses in this field are the application of the KD and its metabolites in treating brain disorders such as epilepsy, the role and mechanisms of the KD and its metabolites in brain metabolism, and the effects of the physiological properties of KD metabolites (e.g., KB) such as antioxidative stress and neuroprotection on brain health.

**Conclusion:**

The KD is beneficial for brain health, and its use in treating brain disorders has garnered widespread attention and recognition globally. This study provides a comprehensive and in-depth analysis of the literature in this field, offering valuable insights into the research hotspots and future directions for investigation.

## 1 Introduction

The Ketogenic diet (KD) is a high-fat, low-carbohydrate diet designed to mimic the metabolic effects of fasting without depriving the body of the essential energy required for growth and development (1, 2). With the implementation of KD, ketone bodies (KB) can replace carbohydrates as the primary fuel for the brain and central nervous system. KB is a collective term for acetoacetate, beta-hydroxybutyrate (BHB), and acetone (3). The use of KD can be traced back to 500 B.C., when it was first employed as a treatment for epilepsy. By the 1920s, physicians had officially adopted KD as a treatment for epilepsy. The term ‘KD’ derives from its ability to elevate circulating concentrations of KB (4). Research in the 1990s reaffirmed the efficacy of KD in managing drug-resistant and pediatric epilepsy, leading to a surge in publications and significant scholarly attention in this domain (5). Presently, KD is a well-established non-pharmacological therapy for refractory pediatric epilepsy (6, 7). Four distinct types of KD have demonstrated clear therapeutic efficacy: the modified Atkins diet, medium-chain triglyceride KD, low glycemic index treatment, and the classic long-chain triglyceride KD (8). It has been shown that KD has potential in the treatment of various neurological disorders such as epilepsy and traumatic brain injury by enhancing *γ*-aminobutyric acid-mediated neurotransmission. For instance, by increasing the expression of cation-chloride cotransporters like KCC2, KD supports the functionality of the gamma-aminobutyric acidergic system, thereby modulating neuronal excitability and offering potential benefits in conditions such as cognitive deficits (9). Recent years have seen a growing interest in KD due to its demonstrated efficacy in treating neurological conditions, including epilepsy, Alzheimer’s disease, and Parkinson’s disease (10). In conclusion, KD is a promising non-pharmacological treatment for a wide range of diseases (11). The role and mechanism of its metabolite KB has been extensively studied and has received much attention from researchers.

During KD, the body becomes deficient in oxaloacetate due to the high-fat, low-carbohydrate diet, which reduces glycolysis and promotes gluconeogenesis through the consumption of oxaloacetate (12). As a result, in the presence of limited oxaloacetate, fatty acids are oxidized by beta-oxidation in hepatocytes, leading to the accumulation of acetyl-CoA. However, hepatocytes direct only a small portion of acetyl-CoA into the tricarboxylic acid cycle, while the majority is channeled into the ketone production pathway, where it is converted into acetoacetate. Acetoacetate is then reduced to BHB and can also undergo spontaneous decarboxylation to form acetone (13, 14). KB plays a crucial role in the human body, serving as a central node in physiological homeostasis and as an important alternative metabolic fuel. It is integral to cellular metabolism, homeostasis, and signaling, providing a primary energy source for extrahepatic tissues such as the brain, skeletal muscle, and heart (12). KB is also closely linked to brain health, not only regulating cerebral metabolism but also exerting neuroprotective effects (15, 16).

KB enters the brain through the blood–brain barrier via monocarboxylic acid transporters, which are regulated by circulating KB levels rather than neuronal activity. Thus, when circulating KB concentrations are elevated (e.g., during a KD), KB replaces glucose as the brain’s primary fuel source. This property makes KB widely recognized for its therapeutic potential in treating conditions characterized by impaired glucose metabolism (17, 18). KB can mitigate symptoms of energy deficiency in neurodegenerative diseases by enhancing brain metabolism (16). Furthermore, the conversion of KB into a utilizable form does not require ATP, and its uptake by the brain occurs more rapidly than that of glucose, thereby improving the efficiency of cerebral energy metabolism. Beyond its role as a fuel, KB enhances brain energy metabolism by increasing the antioxidant capacity of brain cells, reducing mitochondrial dysfunction, and enhancing mitochondrial efficiency (19). Research has demonstrated that KB confers numerous potential protective effects on brain health, including antioxidant, anti-inflammatory, and neuroprotective properties (20). For instance, KB can mitigate oxidative stress by inhibiting reactive oxygen species production, thereby exerting neuroprotective effects by modulating mitochondrial permeability and reducing neuroinflammation (21). Additionally, KB has been proposed to have therapeutic potential in conditions associated with substrate deficiency, insulin resistance, free radical damage, and hypoxia (22). Growing evidence indicates that KB has significant protective effects on brain health and supports its clinical potential. However, further research is required to fully explore and realize its clinical applications (23). Notably, there are more publications in the field, which is not conducive for researchers to get a quick and comprehensive understanding of the field being. And the bibliometric analysis can help to identify the research hotspots and directions in this field, which helps researchers to have a comprehensive and in-depth understanding of the field.

Bibliometric analysis was first conceptualized in 1969 and is now widely used to analyze publications (24). Bibliometrics involves the analysis of published literature and its associated data (e.g., keywords, citations, abstracts, etc.). It studies scholarly publishing through statistical data to describe or show the relationships between published literature (25). Bibliometric analysis allows us to study the evolutionary dynamics of a particular field and helps to understand future research hotspots in that field (26). Additionally, analyzing research hotspots and trends in published literature helps provide a quick overview of the field (27, 28). Although much literature has been published on the link between KD and brain health, no researcher has conducted a bibliometric analysis of this field. Consequently, the research hotspots and trends in this area remain unclear. We addressed this issue by conducting a comprehensive bibliometric analysis of the field related to the link between KD and brain health using R software, VOSviewer, and CiteSpace. We hope that by elucidating the current state of research in this field and analyzing the hotspots and emerging trends, we can provide valuable references for future research in this area.

## 2 Materials and methods

### 2.1 Data collection

Data for this investigation were sourced and downloaded from WoSCC (Guilin Medical University’s subscription version) on July 15, 2024. The search strategy employed was as follows: ((((TS = (“ketogenic diet*”)) AND TS = (Brain or Encephalon)) AND DOP = (2013-01-01/2024-07-15)) AND DT = (Article OR Review)) AND LA = (English) (Note: TS = Topics; DOP=Publication date; DT = Document type; LA = Language). Following the exclusion of irrelevant records, a total of 1,162 documents were obtained without any duplicates. The documents were saved in plain text format and exported as complete records, inclusive of their cited references.

### 2.2 Data analysis

For the bibliometric analysis, this study utilized advanced data visualization and scientific knowledge mapping tools, including Origin 2018, R software (version 3.6.3^1^), VOSviewer (version 1.6.18), and CiteSpace (version 6.1.4). National and institutional co-authorship networks, along with source co-citation and keyword co-occurrence analyses, were visualized using VOSviewer. The specific parameters were: (1) The national co-authorship network included countries with at least 5 publications. (2) The institutional co-authorship network included institutions with at least 5 publications. (3) Source co-citation analysis considered sources with a minimum of 2 citations. (4) Keyword co-occurrence analysis included keywords appearing at least 13 times, with synonymous keywords combined. The impact factors used in this study were obtained from the Journal Citation Reports (JCR) for the year 2023.

## 3 Results

### 3.1 General landscapes of included documents on KD in relation to brain health

**Figure 1:**
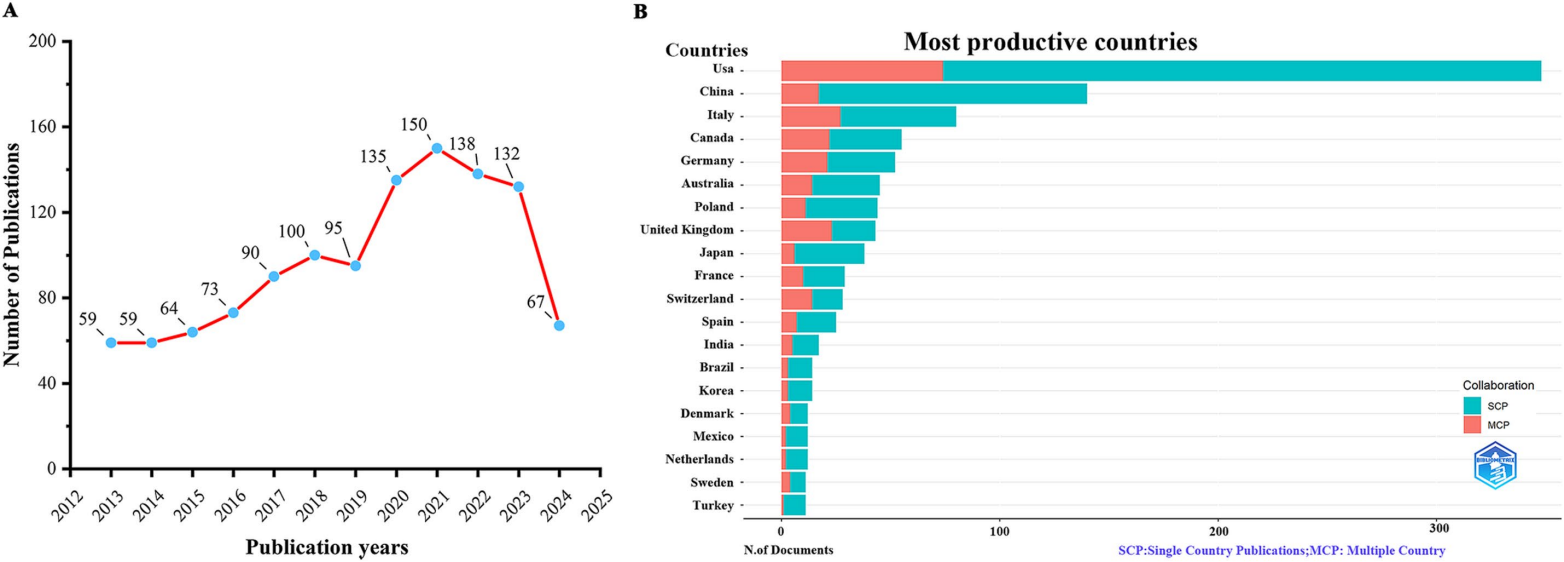
Annual publication trends in the area of the ketogenic diet in relation to brain health, 2013–2024. **(A)** Trends in publication results by year. **(B)** Country and collaboration distribution of corresponding authors.

We collected a total of 1,162 publications from WoSCC. As shown in Figure 1A, the relevant literature in the field of the relationship between the KD and brain health exhibited a general upward trend from 2013 to 2021, reaching a peak of 150 articles in 2021. The largest increase, 40 articles occurred from 2019 to 2020. However, there was a slight decrease in the number of publications after 2021.

**Figure 2:**
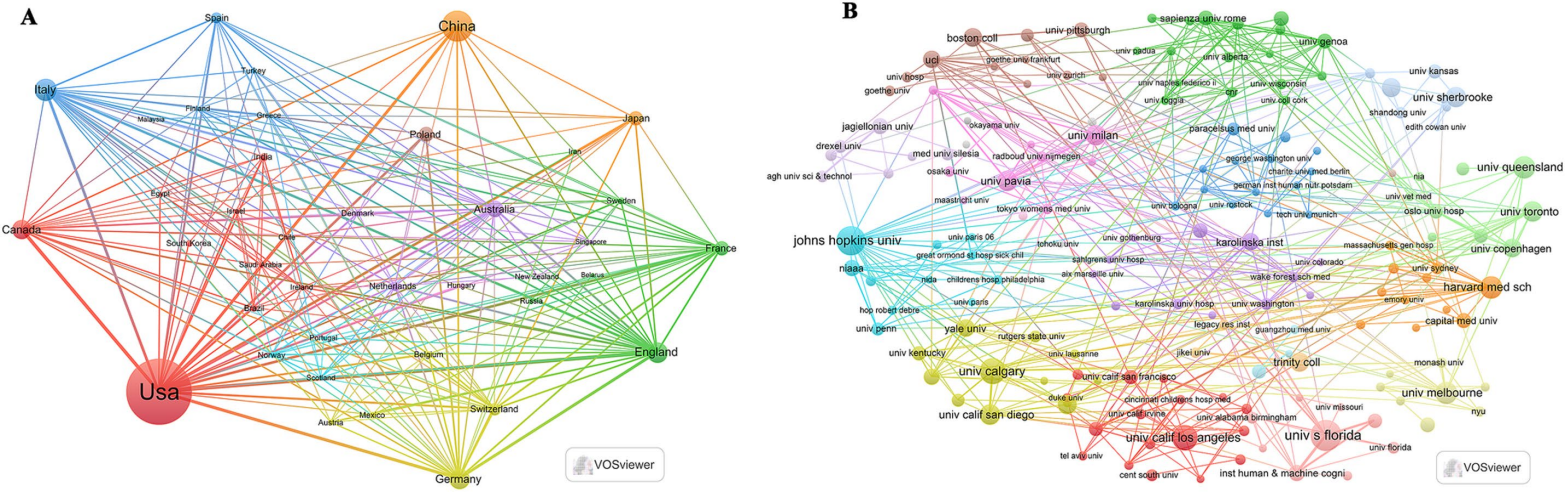
Map of countries/regions and organizations in the field of the relationship between the ketogenic diet and brain health, 2013–2024. **(A)** Map of cooperation between different countries. **(B)** Map of cooperation between different institutions.

As shown in Table 1 and Figure 1B, based on the countries of the corresponding authors, we found that the United States published the highest number of articles with 348 publications. The four countries following closely in the ranking are China (*n* = 140), Italy (*n* = 80), Canada (*n* = 55), and Germany (*n* = 52). Among these five countries with the highest number of publications, Germany has the highest percentage of international collaborations at 40.4%. The lowest percentage of international collaborations was in China, only 12.1%. It is worth mentioning that although the United States has the highest number of publications, its percentage of international collaborations is only 21.3%. Additionally, all five countries collaborate closely with others in the area of the relationship between the KD and brain health. Of these, the United States has the most extensive collaborations (see Figure 2A). As shown in Table 2 and Figure 2B, the University of California System and the University of California, Los Angeles are representative centers of collaboration.

**Table 1:** Corresponding authors in areas related to ketogenic diet and brain health most relevant countries.

Country	Articles	SCP	MCP	Freq	MCP_Ratio
USA	348	274	74	0.299	0.213
China	140	123	17	0.12	0.121
Italy	80	53	27	0.069	0.338
Canada	55	33	22	0.047	0.4
Germany	52	31	21	0.045	0.404
Australia	45	31	14	0.039	0.311
Poland	44	33	11	0.038	0.25
United Kingdom	43	20	23	0.037	0.535
Japan	38	32	6	0.033	0.158
France	29	19	10	0.025	0.345
Switzerland	28	14	14	0.024	0.5
Spain	25	18	7	0.021	0.28
India	17	12	5	0.015	0.294
Brazil	14	11	3	0.012	0.214
Korea	14	11	3	0.012	0.214
Denmark	12	8	4	0.01	0.333
Mexico	12	10	2	0.01	0.167
Netherlands	12	10	2	0.01	0.167
Sweden	11	7	4	0.009	0.364
Turkey	11	10	1	0.009	0.091

SCP, Single Country Publication; MCP, Multiple Country.

**Table 2:** Most relevant affiliations of authors of literature in the field of ketogenic diets and brain health.

Affiliation	Articles
University of California System	108
University of California Los Angeles	63
University of Calgary	61
University of Texas System	48
University College London	46
University of Texas Southwestern Medical Center Dallas	46
State University System of Florida	44
Universite Paris Cite	43
University of London	42
University of South Florida	41
University of California System	39
University of Toronto	37
Goethe University Frankfurt	35
Institut National De La Sante Et De La Recherche Medicale (Inserm)	35

### 3.2 Journals and co-cited journals

**Figure 3:**
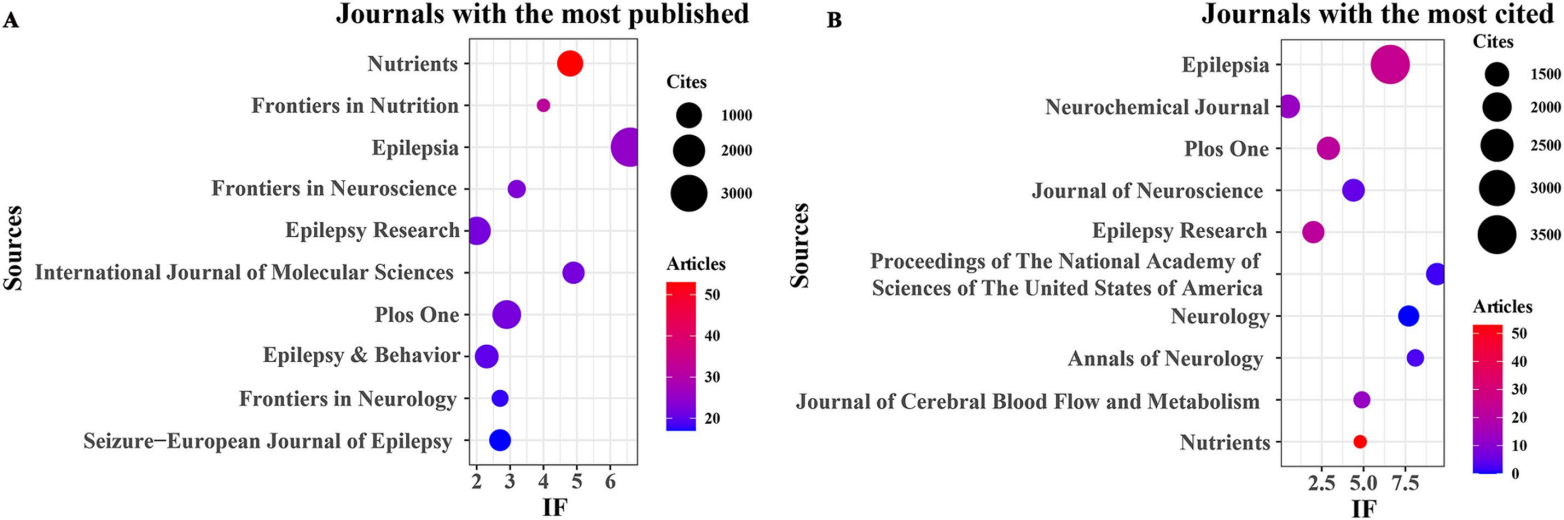
Journals with the most published and journals with the most cited. **(A)** Journals with the most published. **(B)** Journals with the most cited.

**Figure 4:**
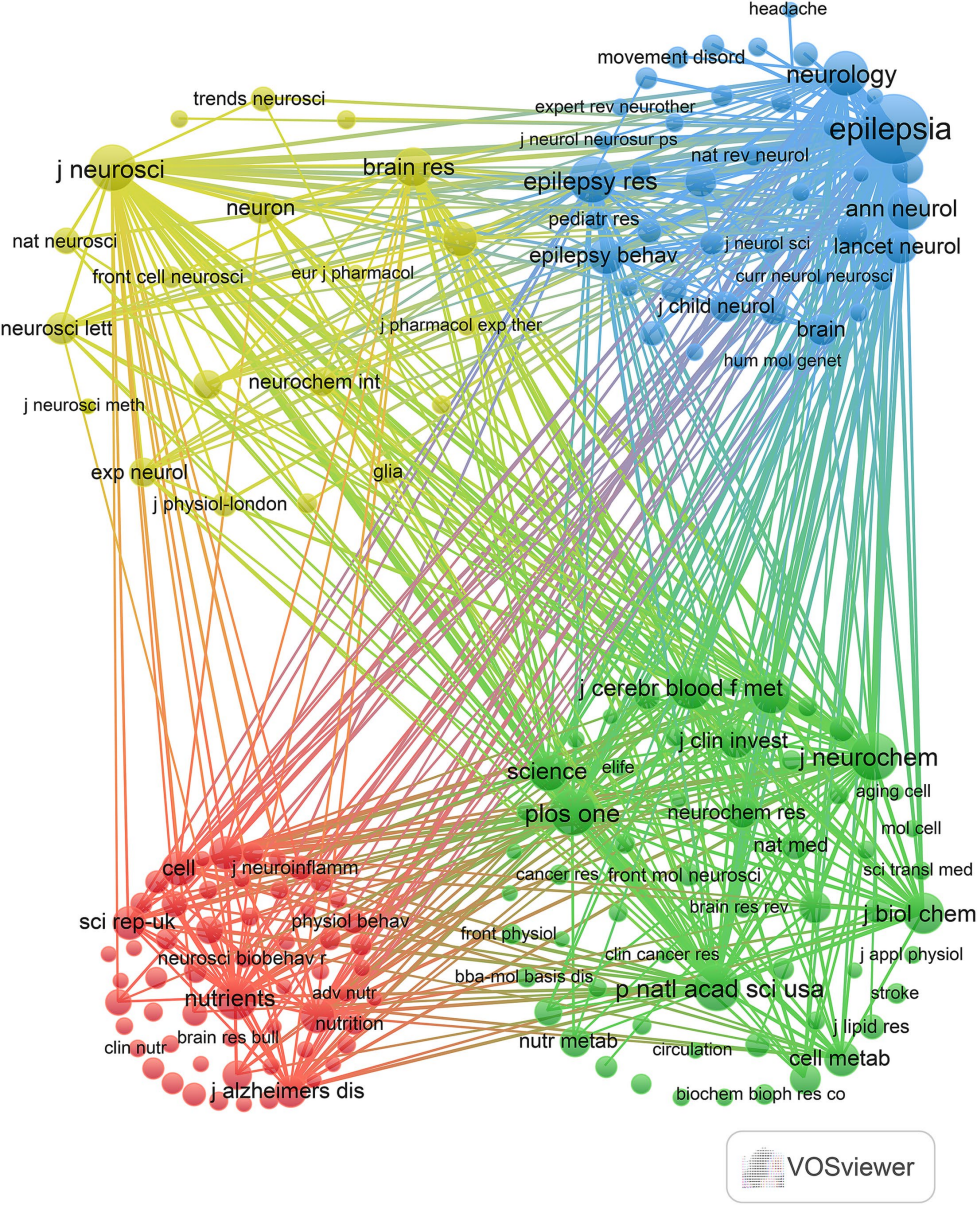
Co-citation journals in the field of ketogenic diets in relation to brain health.

In this study, R software (version 3.6.3) utilizing the bibliometrix and ggplot2 packages was employed to examine the journals and cited journals within the published literature on the KD in relation to brain health. VOSviewer (version 1.6.18) was also utilized to analyze the co-cited journals in this domain. As depicted in Figure 3A and Table 3, the top four journals with the highest number of publications were “Nutrients” (*n* = 53, IF = 4.8), “Frontiers in Nutrition” (*n* = 32, IF = 4), “Epilepsia” (*n* = 25, IF = 6.6), and “Frontiers in Neuroscience” (*n* = 23, IF = 3.2). “Epilepsy Research” (*n* = 22, IF = 2), “International Journal of Molecular Sciences” (*n* = 22, IF = 4.9), and “PLOS ONE” (*n* = 22, IF = 2.9) shared the fifth position. Furthermore, as illustrated in Figure 3B and Table 4, the five most cited journals were “Epilepsia” (*n* = 3,571, IF = 6.6), “Neurochemical Journal” (*n* = 1,442, IF = 0.5), “PLOS ONE” (*n* = 1,411, IF = 2.9), “Journal of Neuroscience” (*n* = 1,368, IF = 4.4), and “Epilepsy Research” (*n* = 1,334, IF = 2). Additionally, the findings in Figure 4 indicate that Nutrients and Epilepsia serve as central hubs of journal collaboration. These results underscore the scarcity of literature published in top-tier journals in this field, highlighting the necessity for further intensive research to achieve significant breakthroughs.

**Table 3:** Top 10 journals with the most published articles.

Sources	Articles	IF	Cites
Nutrients	53	4.8	1,043
Frontiers in Nutrition	32	4	226
Epilepsia	25	6.6	3,571
Frontiers in Neuroscience	23	3.2	343
Epilepsy Research	22	2	1,334
International Journal of Molecular Sciences	22	4.9	621
Plos One	22	2.9	1,411
Epilepsy & Behavior	20	2.3	775
Frontiers in Neurology	18	2.7	289
Seizure-European Journal of Epilepsy	17	2.7	583

IF, Impact factor.

**Table 4:** Top 10 journals with the most cited journals.

Sources	Cites	IF	Documents
Epilepsia	3,571	6.6	25
Neurochemical Journal	1,442	0.5	13
Plos One	1,411	2.9	22
Journal of Neuroscience	1,368	4.4	5
Epilepsy Research	1,334	2	22
Proceedings of The National Academy of Sciences of The United States of America	1,333	9.4	2
Neurology	1,266	7.7	N
Annals of Neurology	1,102	8.1	3
Journal of Cerebral Blood Flow and Metabolism	1,094	4.9	13
Nutrients	1,043	4.8	53

IF, Impact factor.

### 3.3 Most cited references and reference burst

In this study, we analyzed the cited literature in this field using the bibliometrix package of R software and identified the top 20 most cited papers, as shown in Table 5 (19, 29–47). Our research results showed that 1,162 publications from 428 journals were cited. The top three most cited papers are “Sugar for the brain: the role of glucose in physiological and pathological brain function,” “Epilepsy,” and “Tumor-Derived Lactate Modifies Antitumor Immune Response: Effect on Myeloid-Derived Suppressor Cells and NK Cells.” These 20 papers can be categorized into the following topics based on their contents: (1) Application and mechanism of action of the KD in the treatment of epilepsy, Alzheimer’s disease, Parkinson’s disease, and other neurological disorders. (2) Metabolic mechanisms of the KD in normal physiological and pathological states. (3) Mechanisms of the KD on cognitive function and brain health. (4) The KD may cause alterations in gut microbial populations and compositional remodeling. (5) The KD can exert anti-cancer effects by affecting cancer metabolism.

**Table 5:** Top 20 citations related to ketogenic diet and brain health.

Paper	DOI	Total citations	TC per year
MERGENTHALER P, 2013, TRENDS NEUROSCI	10.1016/j.tins.2013.07.001	917	76.42
DEVINSKY O, 2018, NAT REV DIS PRIMERS	10.1038/nrdp.2018.24	554	79.14
HUSAIN Z, 2013, J IMMUNOL	10.4049/jimmunol.1202702	533	44.42
CUNNANE SC, 2020, NAT REV DRUG DISCOV	10.1038/s41573-020-0072-x	434	86.8
NAGPAL R, 2019, EBIOMEDICINE	10.1016/j.ebiom.2019.08.032	319	53.17
COTTER DG, 2013, AM J PHYSIOL-HEART C	10.1152/ajpheart.00646.2012	304	25.33
RAHMAN M, 2014, NAT COMMUN	10.1038/ncomms4944	287	26.09
PRINS M, 2013, DIS MODEL MECH	10.1242/dmm.011585	285	23.75
AUGUSTIN K, 2018, LANCET NEUROL	10.1016/S1474-4422(17)30408-8	271	38.71
SADA N, 2015, SCIENCE	10.1126/science.aaa1299	270	27
STUBBS BJ, 2017, FRONT PHYSIOL	10.3389/fphys.2017.00848	232	29
TALUKDAR S, 2016, CELL METAB	10.1016/j.cmet.2015.12.008	227	25.22
LUTAS A, 2013, TRENDS NEUROSCI	10.1016/j.tins.2012.11.005	221	18.42
GRABACKA M, 2016, INT J MOL SCI	10.3390/ijms17122093	210	23.33
MA D, 2018, SCI REP-UK	10.1038/s41598-018-25190-5	201	28.71
JENSEN NJ, 2020, INT J MOL SCI	10.3390/ijms21228767	184	36.8
NEWELL C, 2016, MOL AUTISM	10.1186/s13229-016-0099-3	182	20.22
ACHANTA LB, 2017, NEUROCHEM RES	10.1007/s11064-016-2099-2	182	22.75
ROGAWSKI MA, 2016, CSH PERSPECT MED	10.1101/cshperspect.a022780	174	19.33
GHOSH S, 2018, GLIA	10.1002/glia.23271	169	24.14

TC, Total citations.

**Figure 5:**
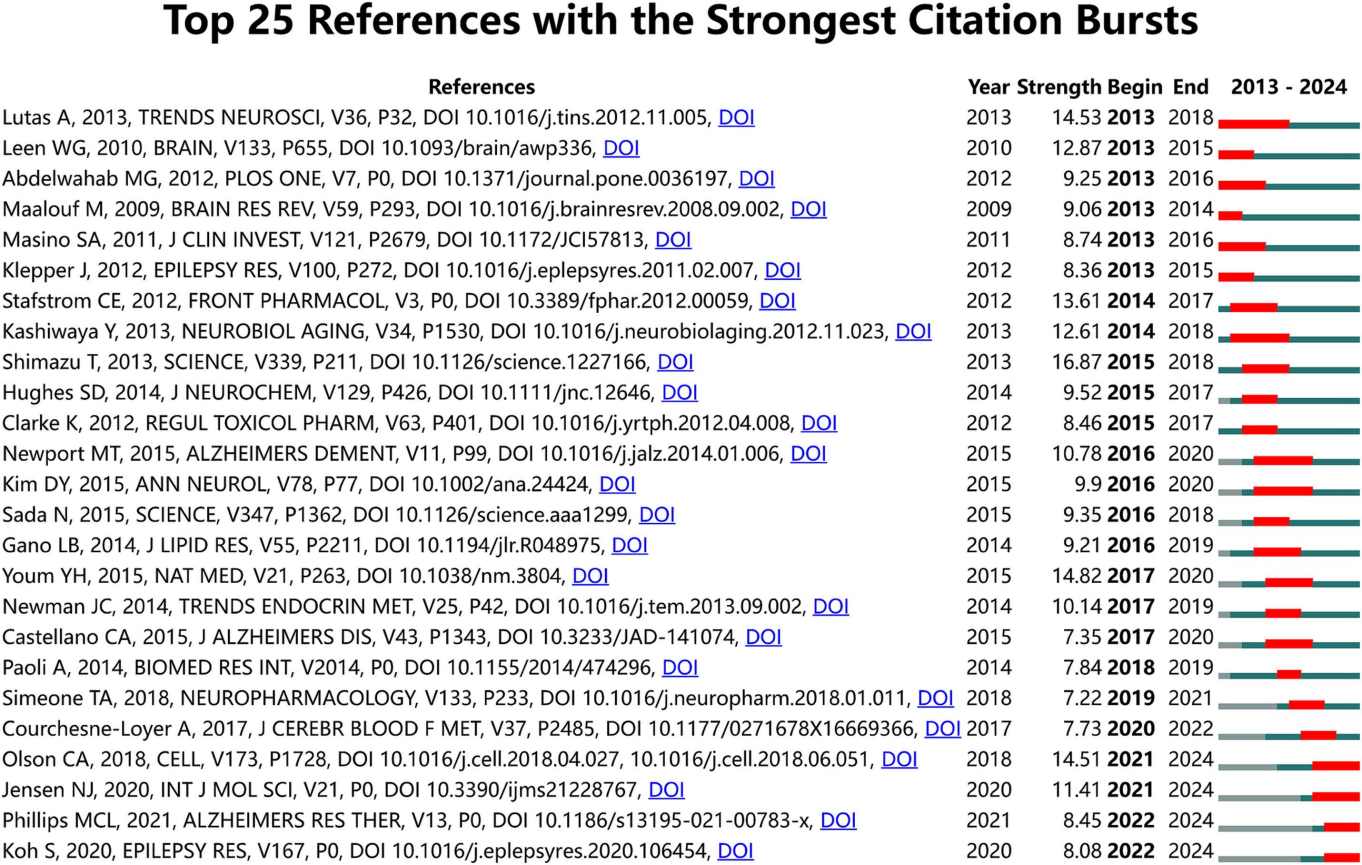
The 25 most cited references in areas related to the ketogenic diet and brain health.

We also used Citespace to analyze citations in the literature on the relationship between the KD and brain health, and the results are shown in Figure 5. The citation burst intensity of the top 25 papers ranged from 7.22 to 16.87. The top - three papers were ‘Suppression of Oxidative Stress by *β* - Hydroxybutyrate, an Endogenous Histone Deacetylase Inhibitor (strength: 16.87)’, ‘Ketogenic diets, mitochondria, and neurological diseases (strength: 14.82)’, and ‘The ketogenic diet: metabolic influences on brain excitation (strength: 14.53)’. These 25 papers can be categorized into the following topics: (1) Anti-inflammatory and neuroprotective effects of the KD and its metabolites such as KB. (2) The role and mechanism of the KD and its metabolites (e.g., KB) in brain energy metabolism. (3) The application of the KD in neurological diseases such as Alzheimer’s disease, epilepsy, and Parkinson’s disease. (4) The KD can exert an antiepileptic effect by modifying the intestinal microbiota to regulate host metabolism and epilepsy susceptibility.

Through the above analysis, we conclude that in recent years there are four research hotspots in the field of the relationship between the KD and brain health: (1) The physiological roles of the KD and its metabolites (e.g., KB), and their therapeutic applications and mechanisms in neurological disorders such as Alzheimer’s disease, epilepsy, and Parkinson’s disease. (2) The role and mechanisms of the KD and its metabolites (e.g., KB) in brain metabolism. (3) The beneficial effects of the KD on brain disorders (e.g., epilepsy) by altering gut microbes. (4) Mechanisms of the anti-inflammatory, neuroprotective, and physiologic effects of the KD and its metabolites (e.g., KB), such as improved cognitive function. However, we also note that there are few clinical studies in this field. Additionally, experimental studies are generally of short duration and small sample sizes, making the long-term effects of the KD on brain health remain unclear. The specific mechanisms underlying the effects of the KD on brain health need to be studied in depth.

### 3.4 Keyword clusters and evolution

**Figure 6:**
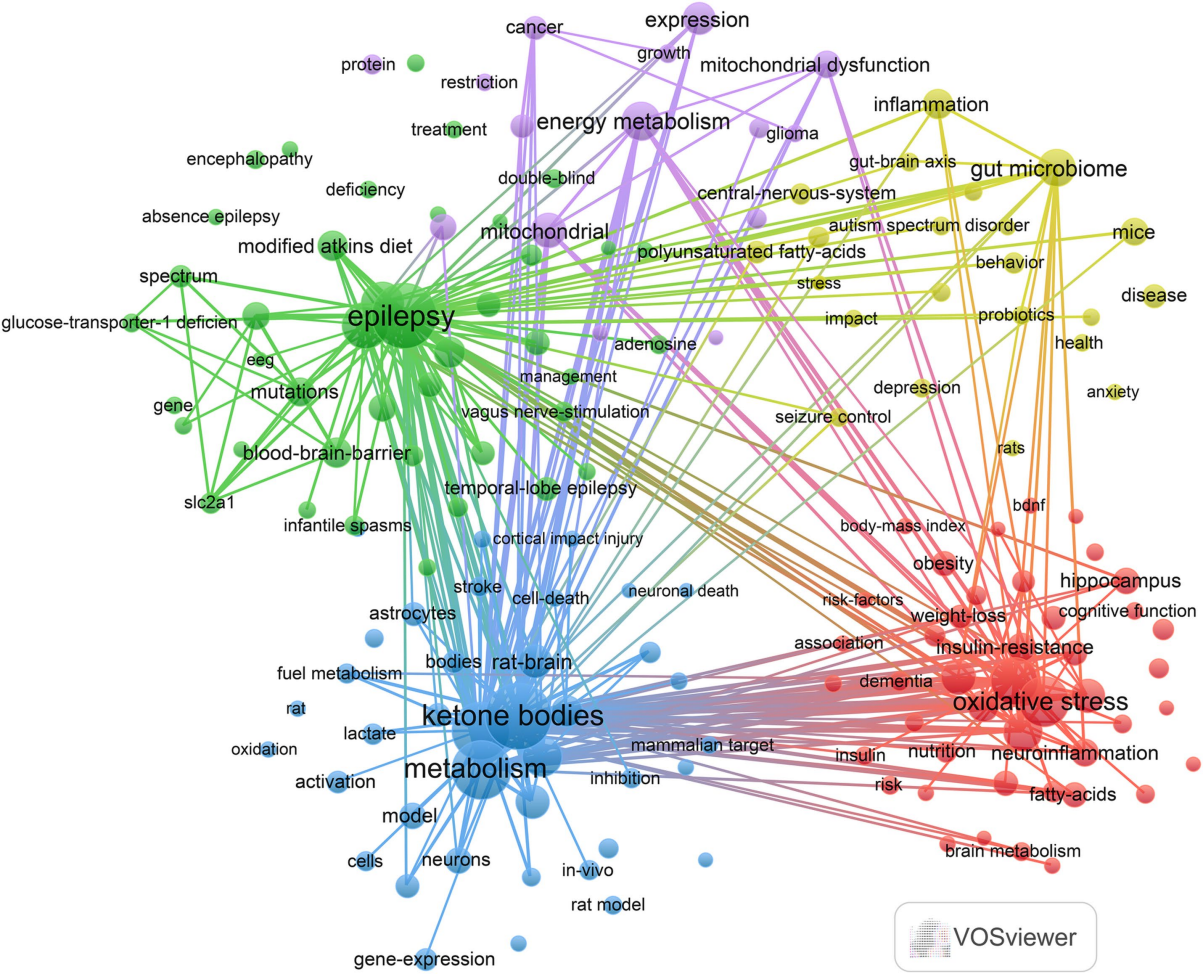
Keyword co-occurrence map of papers in areas related to the ketogenic diet and brain health.

In this study, a keyword clustering analysis of relevant literature on the KD in relation to brain health was conducted using VOSviewer to understand the research trajectory and focus in this field. A total of 5,117 keywords were obtained, of which the top 20 keywords with the highest frequency of occurrence are shown in Table 6. The top 6 keywords in terms of frequency of occurrence were Ketogenic Diet (*n* = 514), Brain (*n* = 213), Beta-Hydroxybutyrate (*n* = 161), Ketone-Bodies (*n* = 157), Metabolism (*n* = 145), Oxidative Stress (*n* = 133). We specified that each keyword appeared ≥13 times, resulting in 162 keywords that were analyzed by clustering, with the results shown in Figure 6. The analysis yielded five different clusters: (1) The impact of the KD and its metabolic processes on brain health and neurodegenerative diseases (red dots): This cluster consisted of 44 keywords, including oxidative stress, cognitive impairment, Alzheimer’s disease, glucose metabolism, and neuroinflammation. (2) Research on the treatment of epilepsy with the KD (green dots): This cluster included 42 keywords, such as epilepsy, modified Atkins diet, glucose transporter-1 deficiency, double-blind, and SLC2A1. (3) Mechanisms of KD in brain metabolism and neuroprotection (blue dots): This cluster contained 39 keywords, including KB, beta-hydroxybutyrate, metabolism, neuroprotection, gene expression, and activated protein kinase. (4) Effect of KD on central nervous system disorders and gut microbiome (yellow dots): This cluster comprised 22 keywords, including gut microbiome, anxiety, autism, central nervous system, and seizure control. (5) Effect of KD on brain cancer and its metabolic mechanisms (purple dots): This cluster included 15 keywords, such as glioma, cancer, energy metabolism, therapy, and Warburg effect.

**Table 6:** Top 20 keywords related to ketogenic diet in relation to brain health.

Rank	Words	Count
1	Ketogenic Diet	514
2	Brain	213
3	Beta-Hydroxybutyrate	161
4	Ketone-Bodies	157
5	Metabolism	145
6	Oxidative Stress	133
7	Children	112
8	Epilepsy	95
9	Mouse Model	88
10	Energy-Metabolism	75
11	Alzheimers-Disease	70
12	Expression	65
13	Glucose	65
14	Rat-Brain	65
15	Blood–Brain-Barrier	53
16	Seizures	52
17	Mutations	50
18	Insulin-Resistance	48
19	Modified Atkins Diet	46
20	Mechanisms	44

**Figure 7:**
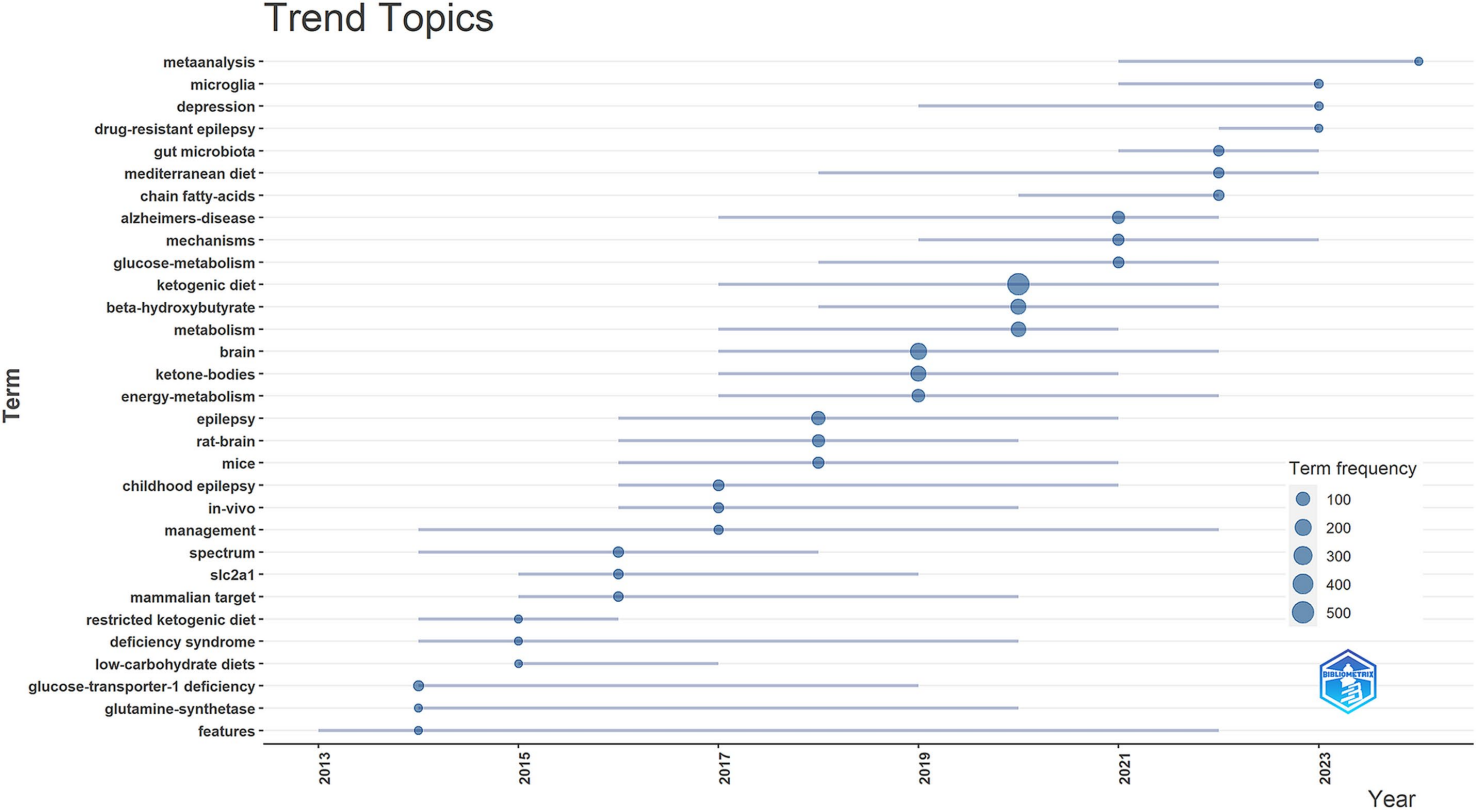
Trending topics in areas related to the ketogenic diet and brain health.

In this study, we used the bibliometrix package in R software to create a trend theme map (see Figure 7). This tool was employed to analyze the temporal development of research themes and the evolution of the field, facilitating an understanding of the research trajectory in this domain. The results show that from 2013 to 2017, the field’s attention has been directed toward studying the application of the keto diet in the treatment of epilepsy, including metabolic disorders that can cause wiring (e.g., GLUT1 deficiency and pyruvate dehydrogenase deficiency). From 2018 to 2024, researchers have shown great interest in the effects of the KD and its metabolites (e.g., KB) on brain metabolism. In recent years, the impact of KD on gut microbes and their beneficial effects on the brain (e.g., antiepileptic effects) by altering the gut microbiota have begun to attract significant attention from researchers.

In summary, through keyword clustering and trend analysis, we identified three research hotspots in the area of the relationship between KD and brain health: (1) the effects of KD and their metabolites (e.g., KB) on brain metabolism; (2) the application and mechanisms of the KD in the treatment of brain diseases, including epilepsy, Alzheimer’s disease, and other neurological disorders; (3) The ability of the KD and its metabolites to attenuate oxidative stress and thus produce neuroprotective effects is also one of the hot topics of research.

## 4 Discussion

### 4.1 General information

In this study, we included 1,162 pieces of literature from 2013 to 2024 and conducted a bibliometric and visual analysis of these works. The results show that the literature published in this field experienced significant growth from 2013 to 2021. Since 2021, there has been a noticeable decline in the number of publications, indicating a downward trend. However, it is important to note that the search cutoff for this study was July 15, 2024, and as such, the data for 2024 is incomplete. Overall, the decrease in publications is relatively modest, suggesting that the observed reduction in research output is likely temporary, and we anticipate that research in this field will resume its upward trajectory in the future. Our analysis suggests the following reasons for this decline: (1) Uncertainty of Adverse Effects: Although the likelihood is small, adverse effects of the KD are possible, including gastrointestinal symptoms, which are most common when the diet is used to treat epilepsy (48). (2) Limited Research Basis: Most studies in this field have small sample sizes, short durations, and are mostly preliminary, resulting in highly limited and non-generalizable findings (49). Current evidence confirming the effectiveness of the KD is mostly indirect, with insufficient direct evidence. There are also fewer available studies in this field (50). (3) Challenges in Clinical Application: While the KD has been proven effective in treating epilepsy, the development of antiepileptic drugs, which are more effective, has led doctors to prefer these drugs over the KD. Although the KD has shown potential in treating neurological brain disorders such as Alzheimer’s disease and Parkinson’s disease, significant challenges remain in its clinical application (10). Given the limited number of clinical studies in this area, understanding the direction of existing research is essential. Through analyses of the most-cited references and citation bursts, we identified the 25 most frequently cited articles in this domain. The research themes addressed in these articles can be classified into four primary categories: (1) Anti-inflammatory and neuroprotective effects of the KD and its metabolites such as KB. (2) The role and mechanism of the KD and its metabolites (e.g., KB) in brain energy metabolism. (3) The application of the KD in neurological diseases such as Alzheimer’s disease, epilepsy, and Parkinson’s disease. (4) The KD can exert an antiepileptic effect by modifying the intestinal microbiota to regulate host metabolism and epilepsy susceptibility.

The United States is far ahead of other countries in the number of publications on the KD in relation to brain health, indicating substantial interest from U.S. researchers. This interest may be related to local dietary habits in the United States. A total of 1,162 publications were published in 35 journals, with Nutrients, Frontiers in Nutrition, and Epilepsia having the highest number of publications and significantly contributing to the field. Epilepsia is also the most cited journal and a key center for journal collaboration, making it a representative journal in this field.

### 4.2 Hotspots and development trends

By examining citation frequency, citation bursts, keyword occurrence rates, keyword clustering analysis, and keyword trends within the literature, we have pinpointed three primary research focal points in the field concerning the KD and brain health. The first focal point is the application and specific mechanisms of the KD and its metabolites in addressing brain disorders such as epilepsy. The second area of focus is the role and mechanisms of the KD and its metabolites in cerebral metabolism. The third research hotspot is effects and mechanisms of Physiological Properties such as anti-oxidative stress and neuroprotection of KD metabolites (e.g., KB) on brain health.

#### 4.2.1 Application and specific mechanisms of the KD and its metabolites in the treatment of brain disorders such as epilepsy

Our comprehensive review of the literature reveals that the KD has been employed for epilepsy treatment since the 1920s, particularly for pediatric epilepsy. Modified dietary regimes, such as the Atkins diet or low-glycemic diet, have broadened the dietary spectrum and spurred further interest in this field (51, 52). Nevertheless, the precise mechanisms through which the KD alleviates childhood epilepsy remain inadequately understood. Current research posits that its efficacy may be linked to disruptions in glutamatergic synaptic transmission, inhibition of glycolysis, and activation of ATP-sensitive potassium channels (41). Evidence indicates that the KD is also effective for refractory epilepsy. The underlying mechanism involves KB, the diet’s metabolites, which furnish a more efficient energy source for brain cells, thereby helping managing seizures and enhancing brain metabolism (37). Additionally, the KD aids in managing seizures in drug-resistant epilepsy when combined with anticonvulsants (46). Thus, the KD proves to be a potent treatment for epilepsy, particularly beneficial for patients with pediatric epilepsy, refractory epilepsy, drug-resistant epilepsy, and other forms unresponsive to conventional antiepileptic drugs, while avoiding their side effects. Additionally, the low cost of KD therapy can benefit patients in resource-poor regions (53). Due to the unclear therapeutic mechanism, further in-depth studies are warranted.

While initially developed for pediatric epilepsy treatment, the KD is now being increasingly applied to other conditions such as Alzheimer’s disease, Parkinson’s disease, and other neurodegenerative disorders (54). Research demonstrates the advantages of the KD in Parkinson’s disease, with the metabolite *β*-hydroxybutyrate acting as a neuroprotectant against the toxicity of 1-methyl-4-phenyl-1,2,3,6-tetrahydropyridine on dopamine neurons, thus decelerating the disease’s progression (55). Substantial evidence suggests that the origins of neurodegenerative diseases like Alzheimer’s disease are linked to impaired glucose energy metabolism in the brain (32). A KD enhances circulating KB, which support brain metabolism during energy deficits and alleviate metabolic disturbances in neurodegenerative conditions (19). Moreover, β-hydroxybutyrate, a KD metabolite, mitigates Alzheimer’s pathology by inhibiting NLRP3 inflammasomes. Given the absence of effective Alzheimer’s treatments, the KD presents new therapeutic possibilities (56). This underscores the significant potential of the KD in managing neurodegenerative diseases such as Alzheimer’s and Parkinson’s diseases.

Nonetheless, it is crucial to acknowledge that the KD and its metabolites exhibit certain limitations in treating brain disorders such as epilepsy. These include: (1) the underlying mechanisms of the KD’s efficacy in epilepsy treatment remain largely unexplored, necessitating further investigation to elucidate these mechanisms (54). (2) Clinical trials are insufficient, with most studies characterized by small sample sizes and short durations, thereby limiting the generalizability and validity of their findings.

#### 4.2.2 Role and mechanisms of the KD and its metabolites in brain metabolism

KB, metabolites produced from the KD, serve as an essential energy source for brain metabolism, particularly during periods of glucose insufficiency (57). Research has shown that when plasma KB levels are moderately elevated, the brain preferentially absorbs and utilizes KB molecules over glucose (58). Numerous studies have established that the onset of various neurological conditions, including several neurodegenerative diseases, is linked to impaired brain energy metabolism, often characterized by diminished glucose metabolism (17, 59). Notably, the brain’s ability to uptake and metabolize KB remains intact, suggesting potential therapeutic interventions involving KB for these diseases (60). In neurological disorders such as epilepsy, Alzheimer’s disease, and traumatic brain injury, KD has demonstrated therapeutic benefits by alleviating hypoglycemia and its associated adverse effects, such as neuronal hyperexcitability and cerebral energy deficits (59).

In individuals with mild cognitive impairment, KB can uncouple respiration and influence histone acetylation, thereby affecting gene expression. Additionally, certain molecules of KB can bypass low cerebral glucose metabolism to supply the brain with necessary energy, thereby enhancing cerebral energy metabolism in patients with mild cognitive impairment (61, 62). Conversely, in Alzheimer’s disease patients, KB compensate for the early-stage mitochondrial energy supply deficiencies. They also enhance glycolysis and the GABA-glutamine cycle. By promoting glycolysis and increasing lactate production, KB facilitate astrocyte-neuron lactate shuttling, thereby providing sufficient energy support to neurons. Furthermore, KB reduce toxic beta-amyloid deposition and prevent its entry into the brain, thus offering therapeutic benefits for Alzheimer’s disease (63). The KD and its metabolites significantly impact brain metabolism under both normal physiological and pathological conditions. However, the underlying mechanisms remain inadequately explored. Therefore, elucidating the mechanisms by which the KD and its metabolites affect brain metabolism represents a promising avenue for future research (64).

#### 4.2.3 Effects and mechanisms of physiological properties such as anti-oxidative stress and neuroprotection of KD metabolites (e.g., KB) on brain health

Substantial evidence indicates that the etiology and pathogenesis of numerous brain disorders, including Alzheimer’s disease, epilepsy, and Parkinson’s disease, are linked to oxidative stress and neuroinflammation (65, 66). Current studies consider chronic inflammation and oxidative stress as pivotal factors in the progression of Alzheimer’s disease (67). Due to the brain’s high energy requirements, it consumes large amounts of oxygen, leading to the production of reactive oxygen species (ROS) and subsequent oxidative stress. Conversely, the primary physiological functions of the KD pertain to enhancing mitochondrial function and mitigating oxidative stress. Among the metabolites produced by the KD, *β*-hydroxybutyrate is one of those that has been extensively studied. Research demonstrates that β-hydroxybutyrate reduces ROS production, thereby alleviating oxidative stress and enhancing mitochondrial function (68).

Current research suggests that the mechanisms by which the KD exerts anti-oxidative stress effects include the following: (1) The KD activates the endogenous cellular antioxidant system by stimulating nuclear factor erythroid-derived 2 (NF-E2)-related factor 2 (Nrf2), which upregulates the transcription of detoxification genes (69, 70). (2) The metabolic expression of uncoupling proteins induced by KB enhances the electron transport chain and decreases mitochondrial membrane potential, thereby reducing ROS production and exerting anti-oxidative stress effects (65). (3) The KD functions as an anti-oxidative stressor by elevating NAD/NADH levels, thereby blocking ROS (71). By reducing oxidative stress, it can protect brain cells and neurons from oxidative stress-induced apoptosis, thus playing a neuroprotective role (72). Furthermore, the KD and its metabolites act as signaling molecules to modulate the glutathione system, preventing cellular damage caused by enhanced oxidative stress induced by seizures (57).

In conclusion, the physiological properties of KD metabolites, such as anti-oxidative stress and neuroprotection, are advantageous for brain health. Their therapeutic applications in neurological disorders have garnered significant attention, highlighting their substantial therapeutic potential in brain diseases (50). However, the complete mechanisms underlying their treatment of these disorders remain incomplete and require further investigation.

## 5 Limitations and future directions

### 5.1 Limitations

Firstly, our sources were exclusively drawn from the Web of Science Core Collection (WoSCC) database, potentially overlooking relevant publications not indexed in WoSCC. Nevertheless, the WoSCC database is widely recognized as a reliable resource for bibliometric analysis. Secondly, this study was restricted to English-language literature, thereby excluding non-English publications, which may impose certain limitations. However, given that English is one of the most universally spoken languages, our study’s coverage of publications in this domain remains comprehensive. Therefore, despite these limitations, our research retains its credibility.

### 5.2 Future directions

The KD has demonstrated significant therapeutic potential in treating brain disorders, evolving from its initial application in epilepsy treatment to addressing other degenerative neurological conditions, and has garnered considerable recognition among researchers. The KD enhances brain energy metabolism and supports brain function. Additionally, its anti-oxidative stress and neuroprotective properties contribute significantly to brain health. Nonetheless, this study highlights that the mechanisms through which the KD treats brain diseases and enhances brain metabolism are not yet thoroughly understood, and its anti-oxidative stress and neuroprotective effects require further investigation. Future research should focus on elucidating the mechanisms by which the KD influences brain metabolism and clarifying its impact on brain health, particularly concerning anti-oxidative stress and neuroprotection. Such insights are essential for the broader and more effective application of the KD in treating brain diseases.

## 6 Conclusion

Our analysis of publications in this field offers researchers a concise and thorough overview, facilitating a clear and comprehensive understanding of the subject. It also provides valuable insights into future research directions within the domain. This study identifies three key research hotspots concerning the KD and brain health:

- Application and Specific Mechanisms of the KD and Its Metabolites in Treating Brain Disorders such as Epilepsy.
- Role and Mechanisms of the KD and its Metabolites in Brain Metabolism.
- Effects and Mechanisms of Physiological Properties such as Anti-oxidative Stress and Neuroprotection of KD Metabolites (e.g., KB) on Brain Health.

## Data Availability

The original contributions presented in the study are included in the article/supplementary material, further inquiries can be directed to the corresponding authors.

## Footnotes

^1^ http://www.bibliometrix.org
